# Sigma Metric Evaluation of Hematological Parameters: A Retrospective Quality Assessment

**DOI:** 10.7759/cureus.91375

**Published:** 2025-09-01

**Authors:** Parth Goswami, Garima Anandani, Vaishali Bhankhodia

**Affiliations:** 1 Pathology, All India Institute of Medical Sciences (AIIMS) Rajkot, Rajkot, IND

**Keywords:** analytical process, hematology, quality control, sigma metric, westgard rule

## Abstract

Introduction: Clinical hematology laboratories play a pivotal role in ensuring accurate diagnosis and optimal patient care. Quality control (QC) is essential for monitoring the analytical phase and detecting errors that may arise due to instrument malfunction, environmental factors, or operator-related issues. Sigma metrics, a statistical tool widely used in clinical biochemistry, offers a more objective measure of analytical performance, though its adoption in hematology is still evolving.

Aims and objectives: (1) To evaluate the internal quality control (IQC) performance of five key hematological parameters using sigma metrics; (2) to determine optimal IQC frequency based on sigma analysis; and (3) to assess the utility of sigma metrics as a comprehensive QC tool in hematology.

Materials and methods: A retrospective cross-sectional observational study was conducted over six months (September 2024 to February 2025) in the hematology laboratory of a tertiary care teaching hospital. Three-level QC materials (L1, L2, L3) were analyzed daily for five parameters: hemoglobin, WBC, RBC, hematocrit, and platelets. Sigma values were calculated using the formula (total allowable error {TEa} values were taken from Clinical Laboratories Improvement Act guidelines):

$\sigma = \dfrac{TE_a - \text{Bias}}{\text{CV}\%}$

Results: Hemoglobin and WBC exhibited sigma values >6, indicating excellent analytical performance. RBC and platelets demonstrated acceptable performance, with sigma values ranging between 4 and 6. Hematocrit showed a marginal sigma value of 3.74, falling between 3 and 4, which suggests the need for improvement in its quality control processes. Notably, none of the analytes recorded a sigma value below 3. These findings reflect an overall satisfactory performance of the hematology parameters, with selective areas requiring enhanced QC strategies.

Conclusion: Sigma metric analysis is a valuable tool for assessing analytical performance in hematology. While high-performing parameters can follow simpler QC protocols (e.g., 13s rule), marginal parameters necessitate stricter multi-rule QC. Overall, adopting parameter-specific sigma-based QC enhances laboratory reliability and efficiency.

## Introduction

The clinical hematology laboratories play a crucial role in contributing to patient well-being and facilitating treatment by delivering high-quality results. The primary goal of quality control (QC) is to expeditiously detect any discrepancies in the laboratory by rigorously scrutinizing the accuracy and consistency of the analytical techniques. Particularly in an analytical procedure, laboratory errors are a major factor influencing the quality of patient treatment or care [1].

In the field of hematology laboratory, the quality control plan typically includes the utilization of internal quality controls (IQC) and an external quality assurance scheme. In IQC, control materials with defined values are run in the analyzer, and Levey-Jennings (LJ) charts are generated. These runs are then assessed using the Westgard rules similar to that done by Mao et al. [2].

Westgard himself has notably stated the necessity to adopt improved metrics that allow for individualization of rules and adherence to standard defect limits that are typically associated with them. If the laboratory uses Sigma metric in IQC routinely, then it can assure the precision of the laboratory as well [3].

The Sigma refers to the standard scale used to evaluate process concerns, specifically in the context of identifying laboratory errors [1]. Sigma measurements are widely utilized in biochemistry laboratories, with a plethora of studies and literature accessible on the subject [4]. The attaining of industrial grade quality performance is still far from achievement. The industrial sectors have been far ahead in enhancing their quality as compared to healthcare [5].

The formula for the sigma metrics incorporates the total allowable errors (TEa), bias, and coefficient of variation (CV%). The primary objective of the laboratory process is to reduce variances, reduce laboratory error, and ensure the safety of the patient by providing accurate results [6].

QC in hematology analyzers is critical to ensure the accuracy and reliability of test results. While most laboratories follow manufacturer or accreditation body guidelines, there is variability in IQC practices. In hematology laboratories, the frequency of IQC analysis directly influences error detection capability and overall resource utilization. Optimizing IQC frequency is essential to balance patient safety, analytical reliability, and cost-effectiveness, yet limited data exist to guide evidence-based recommendations in this domain.

This study aimed to assess the IQC of five hematology analytes using the sigma metric approach, to establish the optimal frequency of IQC based on the results of Six Sigma analysis, and to identify the utility of the sigma tool as a complete quality control in the field of hematology laboratories.

## Materials and methods

The study design entails the implementation of a retrospective cross-sectional observational study over a period of the last six months at the hematology laboratory of a tertiary care hospital and teaching institute. The study was approved by institutional ethics committee of AIIMS Rajkot (Approval No: AIIMS/RAJKOT/6th/IEC/ER/21, dated May 20, 2025).

According to the IQC policy of the laboratory, three-level control material of Bio-Rad (Bio-Rad Laboratories, Hercules, CA, USA) (low L1, normal L2, and high values L3) is run in the Sysmex Six-part Hematology Analyzer (Sysmex Corporation, Kobe, Japan) daily in the morning. The interpretation of the Levey-Jennings chart was conducted on a daily basis, followed by the running of patient samples. We assessed the IQC data for five analytes for this study. The evaluated analytes include red blood cells, hemoglobin, hematocrit, platelets, and white blood cells. The instrument is subjected to routine calibration and maintenance as per our protocol. The IQC datasets were obtained from the laboratory's internal quality control document.

The sigma value is tested using the coefficient of variation (CV), the bias percentage, and the total allowable error (TEa) values. The formula for Sigma is calculated as follows:

$\sigma = \dfrac{TE_a - \text{Bias}\%}{\text{CV}\%}$

In 1974, Westgard introduced the notion of total error (TE) for the first time. A composite measure of the uncertainty of a test result was devised by integrating analytical imprecision and bias, often known as systematic error (SE). The TEa values were obtained on the basis of regulations of the Clinical Laboratories Improvement Act (CLIA) [6].

In laboratory testing, bias refers to the systematic error that causes a consistent deviation of the measured value from the true value. Bias will be calculated from the following formula [1].

$\text{Bias}\% = \dfrac{\text{Mean of all laboratories using same instrument and method} - \text{Lab's mean}}{\text{Mean of all laboratories using same instrument and method}} \times 100$

The CV offers a standardized measure of dispersion, enabling the comparison of variability between datasets with varying units or means. During laboratory testing, a smaller coefficient of variation (CV) signifies more accurate and consistent readings, whereas a larger CV implies a greater degree of variability in the test results.

$\text{CV}\% = \dfrac{\text{Standard Deviation (SD)}}{\text{Mean}} \times 100$ 

Values of standard deviation and laboratory mean will be taken from the machine data [1].

For every five hematological parameters, mean values, SD, TEa, CV%, bias%, and Sigma values were tabulated for low, normal, and high-level control results each month of the study.

## Results

Tables 1 presents the Mean, SD values, CV%, and Sigma values for the five hematological analytes at the low-level control (L1) during the study period from September 2024 to February 2025.

**Table 1:** Quality control data of low-level control (L1) CV: coefficient of variation; TEa: total allowable error

Parameter	Target laboratory mean	Laboratory derived mean	SD%	CV%	TEa	Bias%	Sigma value
Hemoglobin	5.5	5.45	0.0625	1.125	7%	0.909	6.46
Red cell count	2.30	2.28	0.0267	1.175	6%	0.869	4.366
White cell count	3.08	3.10	0.0722	2.325	15%	-0.649	6.17
Hematocrit	16.45	16.27	0.21	1.3	6%	1.0942	3.77
Platelet count	92.75	90.75	5.9	7.0	25%	2.15	3.26

Table 2 presents the Mean, SD values, CV%, and Sigma values for the five hematological analytes at the normal-level control (L2) during the study period.

**Table 2:** Quality control data of normal-level control (L2) CV: coefficient of variation; TEa: total allowable error

Parameter	Target laboratory mean	Laboratory derived mean	SD%	CV%	TEa	Bias%	Sigma value
Hemoglobin	11.8	11.7	0.14	1.175	7%	0.847	5.23
Red cell count	4.27	4.21	0.058	1.425	6%	1.405	3.22
White cell count	6.912	7.055	0.0847	1.50	15%	0.0206	9.98
Hematocrit	34.57	34.25	0.56	1.625	6%	0.925	3.12
Platelet count	231.25	229.5	12.975	7.725	25%	0.755	3.13

Table 3 presents the Mean, SD values, CV%, and Sigma values for the five hematological analytes at the high-level control (L3) during the study period

**Table 3:** Quality control data of high-level control (L3) CV: coefficient of variation; TEa: total allowable error

Parameter	Target laboratory mean	Laboratory derived mean	SD%	CV%	TEa	Bias%	Sigma value
Hemoglobin	15.175	15.1	0.152	1.025	7%	0.494	6.34
Red cell count	5.01	4.965	0.0615	1.225	6%	0.898	4.97
White cell count	16.257	16.722	0.191	1.15	15%	2.860	10.55
Hematocrit	43.55	43.2	0.575	1.2	6%	0.803	4.33
Platelet count	522.75	520.25	14.05	2.675	25%	0.478	9.18

Table 4 shows the average Sigma metric values for all three QC levels (L1, L2, and L3) from September 2024 to February 2025.

**Table 4:** Average Sigma metric values for all three control levels (L1, L2, and L3) from September 2024 to February 2025 L1: low level control; L2: normal level control; L3: high level control

Parameter	L1	L2	L3	Compiled Sigma value
Hemoglobin	6.46	5.23	6.34	6.01
Red blood cell count	4.36	3.22	4.97	4.18
White blood cell count	6.17	9.98	10.55	8.9
Hematocrit	3.77	3.12	4.33	3.74
Platelet	3.26	3.13	9.18	5.19

**Figure 1:**
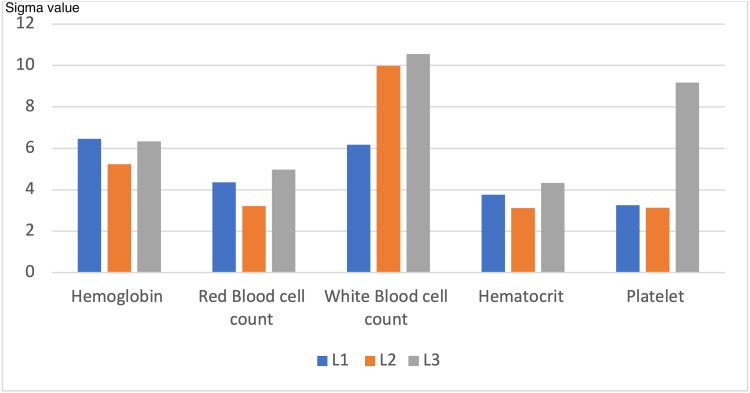
Clustered column chart showing Sigma value comparison for hematology QC parameters across three control levels (L1, L2, and L3) for six month duration QC: quality control; L1: low level control, L2: normal level control, L3: high level control

Figure 1 demonstrates a clustered column chart of Sigma value comparison for hematology QC parameters across three control levels (L1, L2, and L3)

## Discussion

Quality control (QC) plays a vital role in monitoring the analytical phase of laboratory testing within the framework of a quality management system. Its primary objective is to identify, evaluate, and correct potential errors arising from instrument failure, environmental factors, or human error (before patient results are released). Daily internal quality control involves analyzing two or three levels of control materials prior to reporting patient results, ensuring consistency and reliability [7]. Levey-Jennings charts and Westgard rules are routinely applied to interpret control results and guide necessary corrective actions. While parameters like mean, SD, and CV are useful, Six Sigma methodology offers a more robust and comprehensive approach to quality assurance.

The Sigma metric is an analytical tool utilized for evaluating the performance of laboratory testing by quantifying the number of defects per million opportunities (DPM). One sigma denotes 6,90,000 errors per million reports, while six sigma denotes 3.4 errors per million reports [5].

A sigma score of 6 implies that the assay's performance is optimal and no more efforts are needed to improve quality. Assays with a sigma value ranging from 4 to 6 are deemed suitable for their intended purpose, but those with a sigma score between 3 and 4 are regarded as unsuitable [8].

The guidelines for the frequency of internal quality control (IQC) and the evaluation of quality control (QC) based on laboratory results include the following: for >6σ (good tests), a single quality control (QC) test should be conducted each day, alternating between different levels on various days, utilizing a 13s rule; for 4σ to 6σ (suitable for purpose tests), two levels of quality control are required each day, in compliance with the 12.5s rule; in case of 3σ-4σ (underperforming tests), a framework of rules should be established with two tiers of quality control performed twice daily; if <3σ (problems) are identified, the highest standard of quality control is to be executed three times a day at three separate levels [1]. It is recommended to test specimens in duplicate [1].

In our six-month quality control analysis, hemoglobin and white blood cell counts demonstrated excellent analytical performance, each achieving Sigma values above 6. Red blood cells and platelet counts showed acceptable performance, with Sigma metrics ranging between 4 and 6. Hematocrit, however, displayed a Sigma value of 3.74, indicating marginal performance.

The Six Sigma scale ranges from 0 to 6 and can exceed 6 in cases of minimal variability [1]. These findings are consistent with the observations reported by Nagaraj et al., where hemoglobin, WBC, and RBC demonstrated good to excellent performance, while hematocrit (HCT) showed marginal performance, and platelet (PLT) counts exhibited poor performance, indicating the need for stricter quality control for platelets [9]. In comparison, our study also showed good to excellent Sigma values for hemoglobin and WBC, marginal performance for HCT, consistent with Nagaraj et al., but differed in platelet performance, which showed acceptable results, suggesting relatively better analytical control in our setting.

The study by Fuadi reported the lowest Sigma value, below 3, for the HCT parameter [10]. Similarly, in our analysis, Hematocrit also recorded the lowest Sigma value, showing a comparable trend and reinforcing the findings of their study.

The study by Goel et al. demonstrated good to excellent performance for hemoglobin, WBC, and PLT, suggesting that a single level of QC per day using the 1_3_s rule is adequate for these parameters [1]. However, due to the marginal performance of HCT and RBC, a combination of QC rules with two levels of QC conducted twice daily is recommended. Similarly, in our study, HB and WBC exhibited excellent performance, while HCT showed marginal performance, aligning with the findings in this study. 

In the study conducted by Gupta et al., the Sigma values for hemoglobin, WBC, RBC, and PLT were reported to be above 6, while HCT showed a value of 5.6, indicating very good analytical performance across all parameters in their laboratory [11]. This contrasts with our findings, where although HB and WBC demonstrated excellent performance, HCT showed only marginal Sigma levels, and PLT performed acceptably but not excellently. This discrepancy highlights inter-laboratory variability and underscores the need for individualized quality control strategies based on laboratory-specific performance data.

HCT consistently underperformed across our study and previous reports, likely due to higher pre-analytical variability (e.g., sample dilution, plasma trapping) and analytical factors such as instrument methodology and limited sample stability. The study is limited by a relatively small sample size, use of a single hematology analyzer, and reliance solely on CLIA TEa values, which may not align with other regulatory standards. QC frequency recommendations were adapted from Goel et al., principles applicable to hematology analytes [1]. The acceptance of 1_2_s and 2_2_s as warning rather than rejection rules is supported both by our sigma analysis and established Westgard guidelines. Repetition of hematocrit performance findings has been minimized to focus on the unique contribution of this work-sigma metric application to define optimal IQC frequency in a routine hematology setting.

Based on the sigma rules, for all parameters in our study, the 1_2_s and 2_2_s criteria can be accepted as warning rules rather than rejection rules. This means that their occurrence does not necessarily require halting the testing process or repeating the quality control run. Laboratory work can continue without reanalysing the control material unless further rule violations occur.

Based on the findings of our observational study, hemoglobin, WBC, and PLT counts demonstrated good to excellent performance, suggesting that a single level of QC per day using the 1_3_s rule would be sufficient. In contrast, HCT and RBC showed marginal performance, indicating the need for a combination of Westgard rules with two levels of QC to be performed twice daily for more reliable monitoring.

## Conclusions

The present study demonstrates that sigma metric analysis is a useful tool for determining the optimal frequency and selection of Westgard rules for IQC in hematology laboratories. Applying analyte-specific sigma values can help improve resource allocation by tailoring QC frequency to performance, thereby minimizing unnecessary QC runs while maintaining analytical reliability. This approach has the potential to reduce patient risk by ensuring consistent detection of analytical errors. Based on the current findings and supporting literature, reducing QC to a single level is not advisable, as it may compromise error detection, particularly for parameters with marginal sigma performance. Maintaining multi-level QC enhances quality assurance and supports safe, evidence-based laboratory practice.
